# Biological conduits based on spider silk for reconstruction of extended nerve defects

**DOI:** 10.1515/iss-2023-0050

**Published:** 2024-07-19

**Authors:** Peter M. Vogt, Christine Radtke, Nicco Krezdorn, Katja Kollewe, Christina Liebsch, Khaled Dastagir, Sarah Strauß

**Affiliations:** Department of Plastic, Aesthetic, Hand and Reconstructive Surgery and Spider Silk Laboratories, Hannover Medical School, Hannover, Germany; Department of Plastic, Reconstructive and Aesthetic Surgery, Medical University, Vienna, Austria; Department of Neurology, Hannover Medical School, Hannover, Germany

**Keywords:** nerve defects, biological nerve conduits, spider silk, guided regeneration, *Nephila edulis, nerve reconstruction, plastic reconstuction*

## Abstract

**Objectives:**

The availability of appropriate conduits remains an obstacle for successful reconstruction of long-distance nerve defects. In previous sheep trials, we were able to bridge 6 cm nerve gaps with nerve conduits based on spider silk fibers with full functional outcomes. Here, we describe the first application of spider silk for nerve repair in humans.

**Methods:**

Four patients with extended nerve defects (>20 cm) underwent nerve reconstruction by interposition of conduits that were composed of spider silk fibers contained in autologous veins. The longitudinal luminal fibers (approx. 2500 fibers per graft) consisted of drag line silk from *Trichonephila* spiders. All patients were evaluated between 2 and 10 years postreconstruction, clinically, and by neurography.

**Results:**

In all patients, primary wound healing and no adverse reactions to the implanted spider silk material were observed. Patients regained the following relevant functions: protective sensibility, full flexor function with near-normal grasp and powerful function after microvascular gracilis muscle transfer, and key grip function and gross finger flexion after additional tenodesis. One patient with sciatic nerve reconstruction developed protective sensibility of the lower leg, foot, and gait, enabling normal walking and jogging. No neuroma formation or neuropathic or chronic pain occurred in any of the patients.

**Conclusions:**

For patients with extended peripheral nerve defects in the extremities, use of conduits based on spider silk fibers offers the possibility of restoring sensory function and protection from neuroma. This kind of nerve bridges provides new perspectives for the reconstruction of complex and long-distance nerve defects.

## Introduction

Traumatic peripheral nerve injury in industrial countries seems to be quite rare but affect patients significantly. Many of these injuries lead to long-term disability, decreased functionality, and major socio-economic costs. According to the trauma register of the German Society for trauma surgery (Trauma Register DGU^®^), nerve injuries are present in 3.3 % of traumatic injuries of the upper extremities and 1.8 % of the lower extremities [1, 2].

Direct axonal repair is limited to short-distance gaps and may be impaired by scarring and neuroma formation [3, 4]. Extensive nerve gaps, therefore, must be reconstructed with grafts that provide a permissive environment for the axonal outgrowth [5], [6], [7], [8], [9]. Various autologous materials and constructs have been developed and tested over time [10], but most of the approved products are limited to only few centimeters in length [11].

Autologous nerve grafts remain the gold standard as they provide a guiding structure and the necessary microenvironment for axonal outgrowth [12] but are limited in their availability. Additionally, autologous nerve graft donor sites are limited and their harvesting leaves the donor sites asensory. While sural nerve grafts are still the standard of care, their length may be insufficient for large defects while autologous alternatives are mostly paid for with higher donor site sequelae [13]. From the structural point of view, only fresh allografts provide identical nerve graft structure including living Schwann cells. Their disadvantage comprises the use of immunosuppression. Therefore, acellularized allografts are becoming increasingly popular [14, 15]. Alternatively, artificial conduits of different composition and origin can be used without the need of adjuvant therapy or complicated processing. On the biological side, autologous veins with or without muscle fill proved effectiveness in the reconstruction of short distance gaps in humans [16]. The authors reported good results in 85 % of their cases with a minimum follow-up of 14 months in nerve defects ranging from 0.5 to 6 cm in length. They assumed that the results were superior to those reported with other kinds of artificial or biological conduits. Drawback with the usage of acellular nerve grafts and artificial conduits is the size of nerve defect, which can be reconstructed. Yet, no artificial conduit has been demonstrated to be able to reconstruct extended nerve defects larger than 6 cm. In this context, spider silk has been identified as a promising guiding structure for nerve regrowth with negligible adverse effects. Based on these concepts, extensive experimental work and our previously successful study on bridging 6 cm nerve defects in sheep [17], we have continued this work by translational application for bridging of devastating nerve defects of >20 cm in four patients using autologous vein grafts filled with spider silk as a longitudinal guiding material for nerve regeneration and to guide axonal elongation. By this, we observed acceptable engraftment without adverse effects and long-term promising results. To our knowledge, this is the first publication describing the medical application of spider silk as an implant in humans.

## Patients and methods

A total of four patients received vein grafts filled with spider silk due to oncologic or trauma-induced nerve lesions of the extremities. Prior to surgery, all patients had given written consent to the procedure. All procedures were performed as compassionate use due to excessive nerve lesions that could not be bridged by conventional autologous grafts or conduits of that length. All procedures were approved by the Ethical Committee of Hannover Medical School, Hannover Germany.

### Patient 1

**Figure 1:**
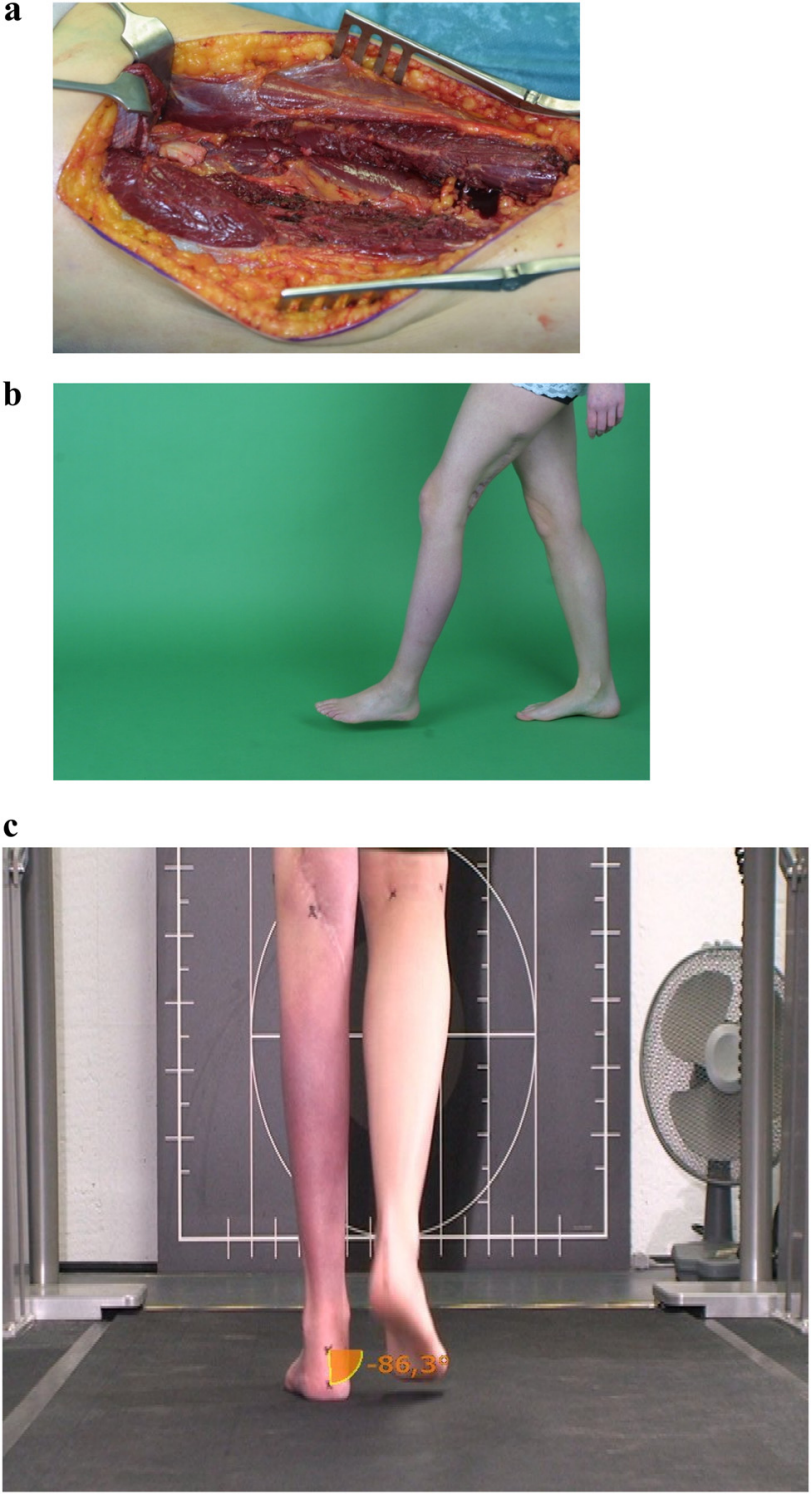
Reconstruction of long sciatic nerve defect. Radical resection of a G2 synovial cell sarcoma of the left sciatic nerve (a). A defect of 23 cm nerve bridging between the proximal sciatic nerve stem and the tibial and peroneal nerve distally. This was accomplished by autologous saphenous vein conduits filled with spider silk fibers (s. video). The patient gained excellent gait (b) in sport physiologic evaluation (c) despite sensory and motoric deficits.

A 26-year-old female patient was referred to our department with an incomplete resection of a G2 synovial cell sarcoma of the left sciatic nerve. Residual tumor at a length of 16 cm required radical resection resulting in a defect of 23 cm between the proximal sciatic nerve stem and the tibial and peroneal nerve distally. After pathology confirmation of complete tumor resection and an otherwise disease-free condition, the defect of the proximal nerve stem and the tibial and peroneal nerve distally was bridged with two autologous saphenous vein conduits filled with 2500 spider silk fibers in each conduit. The postoperative course was uneventful (Figure 1a–c).

### Patient 2

**Figure 2:**
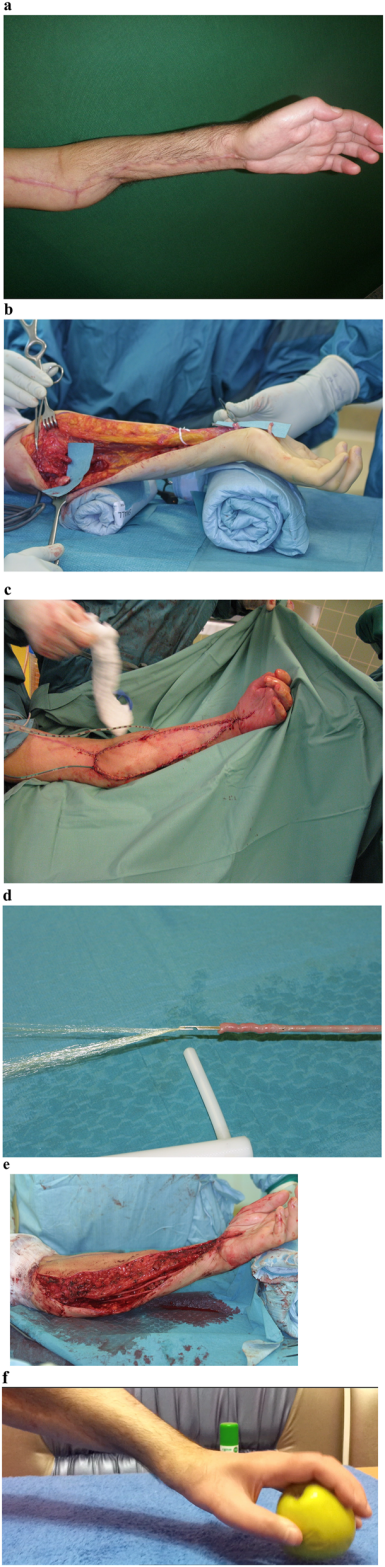
Complex reconstruction of long-distance defects of ulnar and median nerve in the forearm. Total loss of flexor function in forearm and hand after necrotizing infection of the left forearm (a). During extensive dissection, the remnant nerve stumps of the median and ulnar nerve and the distal tendon stumps were identified (b). A free functional ipsilateral myocutaneous innervated (muscle nerve anastomosis to anterior interosseus stump) gracilis flap was used for functional reconstruction of hand and finger flexor function (c). For improvement of protective sensation of the hand, we reconstructed the 25 cm defects of the median and ulnar nerve of the forearm with two 25 cm autologous saphenous long vein/spider silk conduits. Veins were loaded directly from a sterile harvesting rotor (d and e). After additional opponensplasty by an extensor digiti minimi transfer and a split tendon tenodesis of the distal flexor pollicis longus tendon for correction of thumb hyperflexion had been added, the patient gained excellent grip function (f).

A 32-year-old male patient had sustained a total loss of hand function due to resection of all wrist and hand flexor muscles and ulnar and median nerve after necrotizing infection of the left forearm 2 years before in a hospital abroad. We performed extensive tenolysis, neurolysis, and identified the remnant nerve stumps. A free functional ipsilateral myocutaneous innervated gracilis flap was used for functional reconstruction of hand and finger flexor function. The muscle was inserted between the medial epicondylus and the deep finger flexor tendons distally and the flexor pollicis longus tendon. Microvascular reconstruction for revascularization was performed between the flap pedicle and the radial artery end to side and the concomitant vein end to end. The muscle was reinnervated by an end to end coaptation of the muscle nerve branch to the anterior interosseous nerve stump in the forearm. The postoperative course was uneventful and physiotherapy started after 1 week. The patient gradually regained basic function of his hand. However, the patient was not satisfied by the level of functionality and requested improvement. We, therefore, performed a revision by rerouting the muscle and tightening the muscle length. In addition, an opponensplasty by an extensor digiti minimi transfer and a split tendon tenodesis of the distal flexor pollicis longus tendon for correction of thumb hyperflexion was added (New-Zealand procedure). For improvement of protective sensation of the hand, we reconstructed the 25 cm defects of the median and ulnar nerve of the forearm with two 25 cm autologous saphenous long vein/spider silk conduits. After this, the patient had an uneventful postoperative course and continued long-term occupational and physiotherapy in his home country and was evaluated for follow-up after 3 years (Figure 2a–f).

### Patient 3

**Figure 3:**
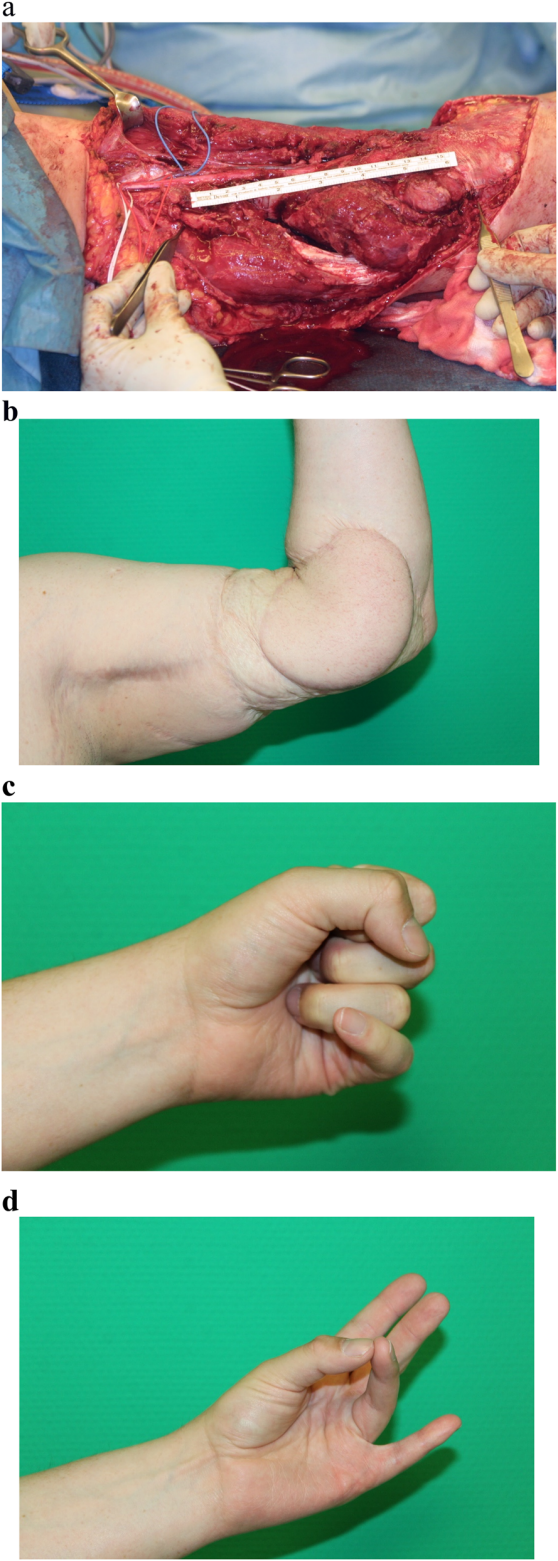
Complex reconstruction of long-distance ulnar nerve lesion in the distal arm and forearm. Avulsion injury of left elbow region and proximal forearm with a third-degree open humeral fracture with a 20 cm long defect lesion of the ulnar nerve (a). Soft tissue reconstruction was accomplished by a free latissimus dorsi flap and reconstruction of the ulnar nerve defect by a saphenous vein/spider silk nerve graft. The postoperative course with excellent elbow flexion and function with full finger flexion and opposition (b–d) 8 years after the operation.

A 26-year-old male patient had sustained an avulsion injury of the left elbow joint and proximal forearm with a third-degree open humeral fracture and a long defect lesion of the ulnar nerve. After serial debridements, the patient received a plate osteosynthesis and soft tissue reconstruction with a free latissimus dorsi flap. In a second step, reconstruction of the 20 cm ulnar nerve defect was achieved by a saphenous vein/spider silk nerve graft. The postoperative course was uneventful and the patient went to physical rehabilitation and was finally followed up 8 years after the operation (Figure 3a–f).

### Patient 4

A 30-year-old male patient had sustained a polytrauma in a car accident and presented with open fractures of both forearms and right lower leg. He required acute revascularization of the avascular left forearm after radial and ulnar artery avulsion with a venous graft. After open fracture fixation, he received a free ALT flap for soft tissue reconstruction of the right leg and a free gracilis flap transfer for functional flexor muscle reconstruction in the arm due to a defect lesion of the flexor pollicis longus and FDS/FDP II-V muscle groups.

**Figure 4:**
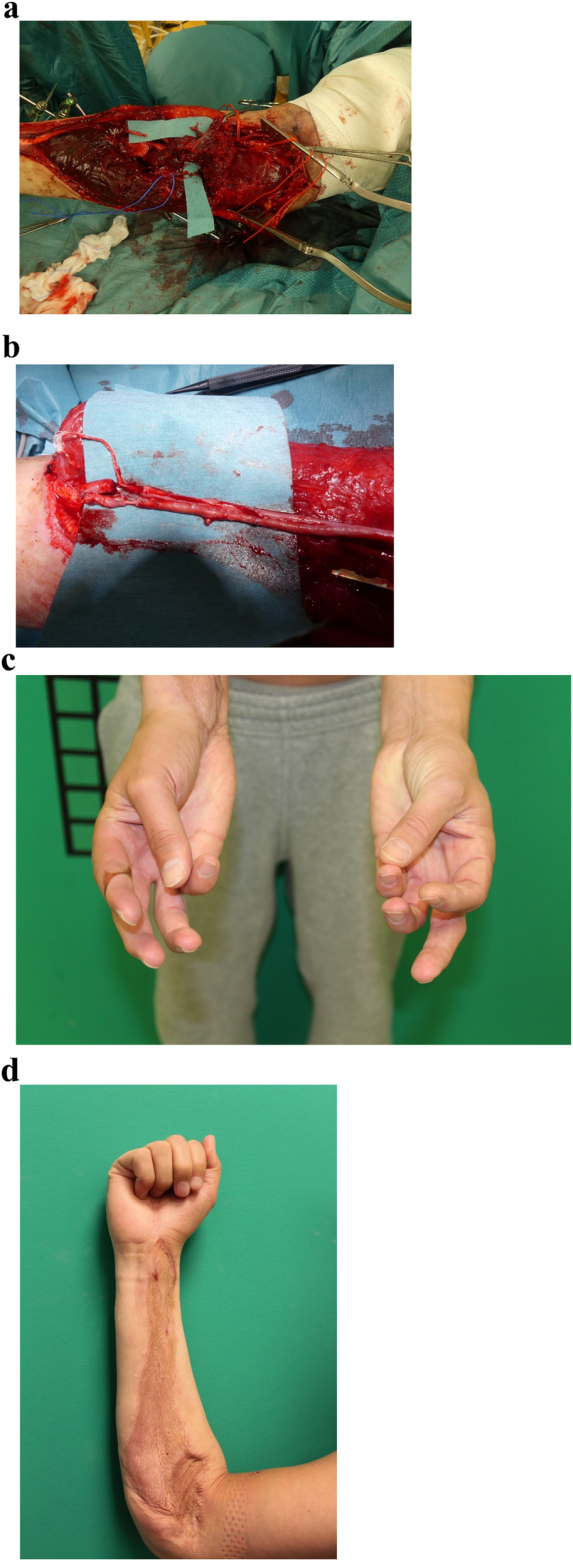
Complex reconstruction of avulsed median nerve in the forearm. Open fractures of both forearms and right lower leg. After open fracture fixation and revascularization of the avascular left forearm after radial and ulnar artery avulsion (a), he received a free ALT soft tissue reconstruction of the right leg and a free gracilis flap transfer for functional flexor muscle reconstruction in the arm due to a defect lesion of the flexor pollicis longus and FDS/FDP II muscle groups. The avulsed median nerve with a 20 cm nerve defect was reconstructed with a saphenous vein/spider silk nerve graft (b). To improve the thumb function, the patient had a secondary brachioradialis to flexor pollicis longus tendon transfer. He gained excellent function with full finger flexion and thumb opposition (c and d).

The avulsed median nerve showed a 20 cm nerve defect and was reconstructed with a saphenous vein/spider silk nerve graft. The postoperative course was uneventful. Due to insufficient thumb flexion, the patient had a secondary operation with an additional secondary brachioradialis to flexor pollicis longus tendon transfer (Figure 4a–d).

### Preparation of nerve grafts

#### Harvesting of vein grafts

Under general anesthesia, an incision anterior of the malleolus medialis up to the knee joint exposed the saphenous vein. After insertion of a blunt cannula distally into the vein and flushing it with saline, the branches were ligated and the vein dissected at the desired length.

#### Insertion of spider silk fibers

For interpositional nerve grafts, autologous saphenous veins harvested from the lower leg were loaded under sterile conditions at the surgical side at the same time of reconstructive surgery with spider silk fibers. Spider silk was sterilized with a standardized protocol.

From each collector, an average of 400 m spider silk fibers on a 30 cm rotor length equaled 2500 fibers per nerve graft. The spider silk loops were transferred into the veins with a surgical thread catcher and the excess length at the vein entrance and exit was trimmed. Veins were oriented in the direction of inflow according to the direction of nerve ingrowth.

All grafts were coapted to the nerve stumps with 8-0 epineural monofilamentous sutures.

#### Neurological evaluation

All patients were evaluated clinically by measurement of muscle activity (electromyography, EMG) and nerve conductivity (electroneurography).

In addition, imaging techniques comprised ultrasound, computed tomography (CT), and magnetic resonance imaging (MRI).

### Spider silk harvesting and processing

#### Animal housing and silk collection

Female *Nephila edulis* spiders were housed freely in a special room brightly illuminated with temperatures around 25 °C and a relative humidity ranging from 40 to 60 %. Spiders were fed with crickets (*Acheta domesticus*) twice a week. Details of keeping and breeding have been described previously by Liebsch et al. [18].

For the process of silk reeling, spiders were collected from their webs by a keeper using disinfected plastic containers and transferred to the reeling station. For silk collection, scientific staff was equipped with sterile gloves, surgical masks, and hairnets. All surfaces including the reeling machine were disinfected with Bacillol AF (Bode Chemie GmbH, Hamburg, Germany). Spiders were handled with live insect forceps (Fine Science Tools GmbH, Heidelberg, Germany) and fixed on a foam pad using sterile 10 × 10 cm gauze swabs (Fuhrmann GmbH, Much, Germany) and pins. A piece of dragline silk always connects the spider to the substrate. By gentle pulling on the thread, it can be collected. For clinical use, silk was reeled on individually designed PTFE (Wiechert-Metall, Garbsen, Germany) collectors. The reeling machines had been developed individually by the Institute for Technical Chemistry (Leibniz Universität Hannover, Hannover, Germany) (Type A machine) and Feuerhahn and Straub [19]. Silk was collected with a maximum speed of 7.2 U/min for 15 min. For each transplant, 300–500 m silk were reeled, meaning use of 10–12 spiders. Afterward, animals were transferred back to their webs, watered, and fed with crickets.

#### Preparation for clinical use

PTFE collectors were placed in individually equipped sterilization containers (Aesculap, Tuttlingen, Germany), wrapped in dust cover bags according to EN 868, ISO 11607, and transferred to the central sterilization facility of Hannover Medical School. Containers with silk on collectors were steam sterilized according to EN 285, ISO 17665 with prevacuum (3 steps, min. 40 mbar absolut), sterilization (134–138 °C, ≥5 min, 3,200–3,400 mbar absolut), and drying (30 min). Afterward, containers were delivered directly to the surgery department.

#### Formal requirements

As spider silk is not a CE certified medical device, declaration of conformity according to German §12 MPG and §7 Abs. Nine MPV had to be signed by the head of department individually for each collector. Declaration of conformity is based on EU directive 93/42/ECC on medical devices. This comprises the declaration that essential requirements listed in 93/42/ECC annex I are fulfilled, as well as risk analysis, a device master record, and others. Medical and care personal was trained on handling and application of spider silk implants yearly.

### Data availability

The data that support the findings of this study are available from the corresponding author upon reasonable request.

## Results

### Patient 1

Ten years after the sarcoma operation, the patient was tumor free and showed sensibility of the saphenous and sural nerve. Clinically neither regeneration of peroneal nerve function nor recovery of foot elevation was observed. The patient showed no phantom pain or neuropathic pain. Despite the lack of functional muscle activity below the sciatic nerve lesion, the patient was very satisfied with the result that enabled her to perform normal daily physical activity and sports. This might be caused by an insidious stiffening of the ankle, which is known to occur after total loss of sciatic nerve function.

Detailed neurological findings did not reveal motoric or sensory nerve function of the tibialis and peroneus below the proximal level of nerve transection.

The electroneurography of the left peroneal nerve (motor), left tibial nerve (motor), and left sural nerve (sensory) showed no responses. Electromyography of the left tibialis anterior muscle (peroneal nerve) and the left gastrocnemius muscle (tibial nerve) revealed pathological spontaneous activity but no volitional activity of motor units indicating complete motor denervation.

### Patient 2

Ten years after the reconstructive surgery, the patient demonstrated key grip function and protective sensibility of thumb and index finger. He did not report any pain or neuropathic pathologies. Long-term neurological and neurophysiologic measurements were not available in the patient’s home country. However, he reported the gain of protective sensibility of the forearm and the palm of his hand and presented functional capabilities via video. Considering the initial disability, the degree of functional gain was significant for the patient.

### Patient 3

Eight years post-trauma, the patient demonstrated full finger and thumb flexion after an additional Zancolli’s procedure had been performed to correct finger claw deformity. Sensory recovery of the dorsal branch of ulnar nerve and protective sensibility of the ulnar nerve were observed.

Motor electroneurography of the left median nerve showed a normal conduction velocity and a reduced amplitude (5.0 mV) of the compound muscle action potential. The same nerve showed a normal amplitude of the sensory potential, but a reduced conduction velocity (42 m/s). Motor and sensory electroneurography of the left ulnar nerve showed no responses. In the electromyography of the left flexor carpi ulnaris muscle pathologic spontaneous activity was found, and motor unit potentials revealed elevated amplitudes but no polyphasicity. The electromyography of the abductor digiti minimi muscle revealed pathological spontaneous activity but no volitional motor unit activity indicating complete motor denervation.

### Patient 4

Thirty-one months after trauma, the patient showed full finger and thumb function after rehabilitation and occupational therapy including protective sensibility of median nerve innervated fingers.

Motor electroneurography of the right median nerve showed a very low amplitude of the compound muscle action potential (<0.2 mV). Testing of the sensory fibers of this nerve showed no response.

A tabular overview is given in Table 1.

**Table 1:** Details of the patients receiving spider fiber–based nerve grafts.

Pat.Nr.	Age at operation (years)/sex	Operation	Procedure	Diagnosis	Follow-up (years)	Results	
						Sensibility	Motor function
1.	26 f	2011	Immediate reconstruction of 23 cm sciatic nerve defect; double graft (saphenous vein/spider silk): sciatic stem to tibial and peroneal nerve	Synovial cell sarcoma of left sciatic nerve	2021 (10)	No regeneration of sensibility; no pain was reported and no clinical evidence for neuroma development	No regeneration of peroneal nerve function
2.	32 m	2011	Late secondary reconstruction of 25 cm median and ulnar nerve defect; (saphenous vein/spider silk) Interposed in median and ulnar nerve graft Functional free gracilis muscle For forearm flexor function, opponensplasty with EDM transfer	Total loss of left forearm and hand function due to resection of all wrist and hand flexor muscles and ulnar and median nerve	2018 (7)	Regeneration of protective sensibility of thumb and index finger; no pain was reported and no clinical evidence for neuroma development	Regeneration of key grip function
3.	26 m	2013	Reconstruction of 20 cm ulnar nerve defect; (saphenous vein/spider silk) Interposed in ulnar nerve. Soft tissue reconstruction by free latissimus flap	Avulsion injury of left arm with third degree open humeral fracture; defect lesion of ulnar nerve	2021 (8)	Sensory recovery of dorsal branch of ulnar nerve, protective sensibility of ulnar nerve; no pain was reported and no clinical evidence for neuroma development	No motor function recovery; full finger and thumb flexion after Zancolli’s procedure
4.	30 m	2019	Reconstruction of 20 cm median nerve defect; (saphenous vein/spider silk) Interposed in median nerve; functional free gracilis muscle For forearm flexor function Secondary brachioradialis to flexor pollicis longus tendon transfer	Polytrauma with open fracture of both forearms and right lower leg; ALT free flap reconstruction of extremities; defect lesion of median nerve and flexor pollicis longus and FDS/FDP II; revascularization of left forearm	2021 (2)	Regeneration of protective sensibility of median nerve innervated fingers; no pain was reported and no clinical evidence for neuroma development	Regeneration of full finger and thumb function

## Discussion

Long-distance defects of major nerves result in significant functional impairment of the patient and pose a major challenge to the reconstructive surgeon. Direct nerve repair in distal nerve transection may lead to short distance neurotization of target muscles or sensory areas and is, therefore, most successful in those short-distance gaps. It may be impaired by scarring and neuroma formation. Extensive nerve gaps must be reconstructed with grafts that provide a permissive environment for axonal outgrowth. Various materials and constructs have been developed and tested over time.

The four cases presented here have been the first clinical applications of spider silk in the context of peripheral nerve defects. The cases are diverse and reconstructions have been complex, as such statistical differences cannot be derived. A standardized clinical model is, therefore, of high relevance. The sural nerve biopsy model described by our group could serve as an interesting option for future evaluation of spider silk application in human patients [20, 21]. One of the limitations of our case series is clearly the dimensions of the defects and lack of controls such as matching autografts, allografts, or artificial conduits. In the individual situations, these options were not available. The excellent results of the animal study [17] on large defects warranted their application in humans for the first time.

Autologous nerve grafts are the gold standard as they provide a guiding structure and generate the necessary microenvironment for axonal outgrowth especially Schwann cell and non-Schwann cell associated metabolism and indirect cytokine action [5], [6], [7], [8], [9]. However, autologous nerve graft donor sites are limited in length and diameter and their harvesting leaves asensory donor sites. Different strategies for their use such as inclusion in flaps or avoiding third party donor sites have been addressed [13, 22], [23], [24], [25]. While sural nerve grafts are still the best standard of care, their availability is limited in large defects and their use mostly paid for with higher donor site sequelae [13]. From the structural point of view, fresh allografts provide identical nerve graft structure including living Schwann cells. In order to achieve engraftment, fresh cadaver nerve allografts would require relevant dosages of immunosuppression, which would affect the recipient – as low dosage regimens were not sufficient to protect the sensitive Schwann cells [26].

Therefore, acellularized or processed allografts are becoming increasingly interesting. Experimentally cold-stored nerve allografts showed functional recovery similar to autografts in the reconstruction of a 3-cm motor nerve defect in rabbits. By contrast, frozen allografts significantly impaired functional recovery and the authors suggested to avoid them [27]. Better results in context of treatment of small defects seem to be achieved using a nonimmunogenic allograft, which had been processed with a combination of irradiation, detergent application, and degradation [28].

A systematic review compared outcomes of autografts, allografts, and conduits. The latter showed the least rate of recovery of sensitivity and the highest rate of complications [29]. Another detailed overview about various approved products, outcome, benefits, and limitations is given in Kornfeld et al. [11] and [30].

In reconstruction of small sensory nerves by an allograft of 3 cm after excision of atraumatic neuroma in two out of four patients, both recovery of pain and sensation were observed. Of note is the fact that neuroma or neuropathic pain did not occur in any of the four patients. Also no complications such as extrusion or infection occurred as described elsewhere [31]. This is important as the diameter of the damaged nerves was significantly different from larger nerve stems of the sciatic, median, and ulnar nerve, respectively, and exposed to direct mechanical irritation.

Bulstra et al. investigated functional outcome after reconstruction of 3 cm long nerve gaps in rabbits by decellularized nerve allografts. Longitudinal ultrasound measurements provided evidence that the cold-stored allograft provided earlier regeneration than the frozen-stored allograft [27]. Further, Farber et al. [32] demonstrated that increased graft length is associated with longer vascularization time of the graft, which also affects the functional result.

In conclusion, all these data do not tackle very long-distance defects as encountered in our patient series.

As an alternative to biological substitutes, artificial conduits of different composition and origin can be used without the need of adjuvant therapy or complicated processing. They provide the promise for off-the-shelf availability. On the biological side, autologous veins with or without muscle fill proved effectiveness in the reconstruction of short-distance gaps in humans [16]. The authors reported good results in 85 % of their cases with a minimum follow-up of 14 months in nerve defects ranging from 0.5 to 6 cm in length. They assumed that the results were superior to those reported with other kinds of artificial or biological conduits. Looking at developments of the last years, numerous strategies have been developed including application of growth factors and nanoparticles to stimulate peripheral nerve regeneration [29]. Results are promising, but none of these is able to treat large defects successfully.

In this study, we present data on bridging of devastating nerve defects of larger than 20 cm in four patients with vein grafts filled with spider silk as guiding material for nerve regeneration. The long-term functional outcomes of up to 10 years (patient 1) showed excellent engraftment without adverse effects. The use of this new technology was justified by the lack of appropriate autologous or allogeneic grafts and the time constraints required to achieve a timely nerve bridging.

The fact that direct reinnervation of muscle activity was achieved in none of our patients must be primarily attributed to the fact that the dimensions of the defects had either destructed significant muscle mass directly or motor endplate loss leading to long-term denervation.

Therefore, the reinnervation of major muscle groups is a challenge that has been best addressed by immediate and direct reinnervation without grafts using appropriate donor nerves such as in brachial plexus surgery. These options are not readily available in cases of large peripheral (sensory and motoric) nerve defects.

However, loss of muscle groups by direct trauma can be sufficiently addressed by free muscle transfer and direct neurotization as demonstrated in our series. The results indicate that the length of axonal growth of up to 80 cm through a guiding structure containing spider silk fibers requires more than just a conduit. Additional Schwann cell population appear to be necessary although in our previous experiment in sheep 6 cm gaps with short distances to the neural plates could be successfully addressed by this approach without additional Schwann cells [17, 33, 34].

In conclusion, long-distance nerve defects remain an ongoing challenge for reconstruction but promising solutions are on the rise. We propose spider silk as an option for reconstruction due to the ease of production, the biological properties, and successful engraftment – even though significant reinnervation was not observed. Future investigations will be directed toward inclusion of Schwann cells for an axon-enhancing environment as well as improved vascularization.
